# The Relationship between Folic Acid and Risk of Autism Spectrum Disorders

**DOI:** 10.3390/healthcare2040429

**Published:** 2014-10-23

**Authors:** Yasmin Neggers

**Affiliations:** Department of Human Nutrition, University of Alabama, Box 870311, 504 University Blvd, Tuscaloosa, AL 35487, USA; E-Mail: yneggers@ches.ua.edu; Tel.: +1-205-348-4706; Fax: +1-205-348-2982

**Keywords:** micronutrients, folic acid, autism, metabolism, genetic, epigenetic

## Abstract

There is considerable scientific evidence that many aspects of diet influence the occurrence of human disease. Many factors such as genetic, psychological, environmental and behavioral characteristics influence development of human disease, and there is a close relationship between nutrition and disease. Though typical Western diets are not overtly deficient in essential nutrients, nutriture of a few micro nutrients such as folic acid has been reported to be sub-optimal, particularly in women of childbearing age. The role of folic acid in the prevention of macrocytic anemia and neural tube defects is well established. However, the relationship between folic acid and risk of autism is still evolving. Furthermore, environmental as well as nutritional factors such as folic acid are now well acknowledged as interacting with the individual genetic background in development of several diseases. In this article, recent research regarding the relationship between folic acid and risk of autism is evaluated.

## 1. Introduction

Autism, also referred to as autistic spectrum disorder (ASD) and pervasive developmental disorder (PDD), defines a group of neurodevelopmental disorders affecting approximately 1% of the population which is usually diagnosed in early childhood [1]. Since there are no definitive biological markers of autism for a majority of cases, diagnosis depends on a range of behavioral signs. Experts disagree about the causes and significance of the recent increases in prevalence of ASD [2]. Despite hundreds of studies, it is still not known why autism incidence increased rapidly during the 1990s and is still increasing in the 2000’s [3]. The findings from updated (March 2014) population-based estimates from the Autism and Developmental Monitoring Network Surveillance (ADDM) in multiple U.S. communities, as reported by the Centers of Disease Control and Prevention (CDC), indicates an overall ASD prevalence of 14.7 per 1000 (95% C.I. = 14.3–5.1) or one in 68 children aged 8 years during 2010 [4]. This latest prevalence estimate of ASD as one in 68 children aged 8 years, was 29% higher than the preceding estimate of one in 88 children or 11.3 per 1000 (95% C.I. = 11.0–11.7) [4].

Both genetic and environmental research has resulted in recognition of the etiologic complexity of ASD. An integrated metabolic profile that reflects the interaction of genetic, epigenetic, environmental and endogenous factors that disturb the pathway of interest needs to be evaluated [5]. Though it is established that ASD is a multi-factorial condition involving both genetic and a wide range of environmental risk factors, only during the past decade has the research into environmental risk factors grown significantly [6]. The contribution from environmental factors was originally thought to be low partly due to high monozygotic twin concordance in earlier studies and partly due to a limited understanding of gene-environment interactions [7]. Over the past 10 years, studies with biological plausible pathways, focused on critical time periods of neurodevelopment have resulted in promising risk and protective factors. One such area of research concerns potentially modifiable nutritional risk factors. Despite a number of studies evaluating the diet and nutritional status in children affected with ASD, there is still a paucity of research directly investigating the association between maternal nutrition and risk of ASD in the offspring [6,7]. Maternal nutrition is essential to fetal brain development, and maternal nutrient deficiencies have been associated with significant increased risk of various adverse neurodevelopmental outcomes, including neural tube defects and schizophrenia [8]. Fetal brain development in terms of structure and function has been shown to be influenced by maternal nutrient balance and deprivation, particularly common during pregnancy due to increased metabolic demands of the growing fetus, as well as increased nutrient needs of maternal tissues [9,10]. Therefore, it is quite plausibile that maternal nutritional status before and during pregnancy may influence ASD risk.

In this article the association between maternal folic acid intake, including folic acid supplementaion and risk of ASD in the offspring will be evaluated. It is of interest that though there is some evidence that folate intake during pregnancy decreases the risk of ASD [8,9,10], several investigators have speculated that a high maternal folate intake due to folic acid fortification of foods may be linked to increased prevalence of ASD [11,12,13,14].

## 2. Folate Metabolism

**Figure 1:**
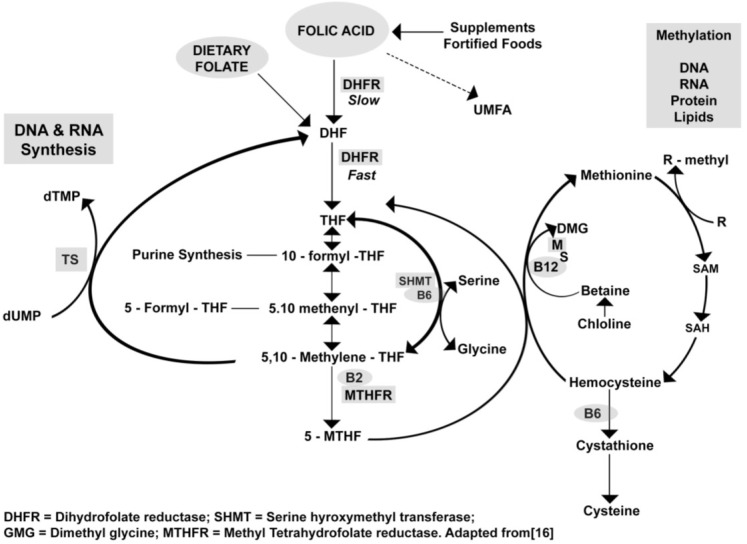
Folate metabolism [16].

Folate is a generic term for a vitamin, which includes naturally occuring food folate (reduced form, largely polyglutamated 5-methyltetrahydrofolate) and folic acid (oxidized form, pteroyl-L-monoglutamic acid) in supplements and fortified foods [15]. Folate has many coenzyme roles that function in the acceptance and transfer of 1-C units. The function of folate in mammals is to aquire single-carbon units, usually from serine, and transfer them in purine and pyrimidine biosynthsis; hence folate coenzymes are essential for synthesis of DNA [11,15]. Folate coenzymes are also necessary for de novo methionone synthesis and several other cellular components. Figure 1 illustrates how the folate cycle facilitates nucleic acid synthesis and is responsible for transfer of 1-C methyl groups to DNA and proteins [16]. Methyl groups added onto cytosine residues in the promoter region CpGs in genomic DNA are central to regulation of gene expression [17,18]. Recent investigations have led to suggestion that children with autism may have altered folate or methionine metabolism resulting in hypotheses that the folate-methionine cycle may play a key role in the etiology of autism. Main *et al*. conducted a systematic review to examine the evidence for the involvement of alterations in folate methionine metabolism in the etiology of autism [18]. The findings of the review of studies reporting data for metabolites, interventions or genes of the folate-methionine pathway and their related polymorphism were conflicting [18]. There was suggestion that changes in concentrations of metabolites of the methionine cycle may be driven by abnormalities in folate transport and/or metabolism. Most genetic studies lacked sufficient power to provide conclusive genetic relations. These investigators concluded that further research is needed before any definitive conclusions can be made about the role for a dysfunctional pathway in the etiology of autism.

Though an evaluation of the entire metabolic pathway of folic acid metabolism along with various genetic varitions in enzymes involved in the numerous pathways will provide greater mechanistic insights in the ASD pathology, it is beyond the scope of this article to do so. A targeted approach focusing on the role of folic acid in DNA methylation and recent epigenitic evidence will be discussed.

## 3. DNA Methylation and the Epigenetic Role of Folic Acid in ASD

Coenzyme 5-methy tetrahydrofolate polyglutamate (5-CH_3_THFR) is needed by the salvage pathway to convert homocystiene to methionine [15]. Methionine can be converted to S-adenosyl methionine (SAM), a molecule with many roles, including methylation of cytosine residue in DNA and of arginine and lysine residues in histones, both of which are involved in regulating gene expression [13]. In the mammalian genome, methylation only occurs on cytocine residues that occur 5’ to a guanosine residue in a CpG dinucleotide. CpG dinucleotides are enridched in “CpG islands” which are found proximal to promoter regions of about half the genes in the genome, and are primarily unmethylated. Methylation of promoter related Cpg islands can supress gene expression by causing chromatin condensation. DNA methylation is important as an epigenetic determinant of gene expression, in the maintainence of DNA integrity and in development of mutations [19]. Errors in normal epigenetic processes are called epimutations, and result in epigenetic silencing of a gene that is not normally silent. Low folate status is often associated with impairment of DNA methylation, but sometimes it leads to hypermethylation and could affect gene expression in complex ways (-19). From these studies, it is not clear whether an excess of folate might have adverse effects on these mutations.

There is speculation that folic acid supplementation may be associated with some aberrant conditions in children [18]. Junaid *et al*. reported that exposure of lymphoblastoid cells to folic acid supplementation causes widespread changes in gene expression [13]. Furthermore, Barua *et al*. [16] suggested that the occurrence of such epigenetic changes during gestational development may impact methylation status of DNA in the offspring’s brain and cause altered gene expression, and since gestational development involves a highly orchestrated regulation of gene expression, this gene dysregulation may affect brain development and may result in various neuropsychiatric conditons. Baura *et al*. [17] identified substantial differently methylated regions (DMRs) in the the cerebral hemispheres of mice offspring of mothers on high folic acid intake as compared to offspring of mice on low maternal folic acid diet. These results support the findings of numerous studies that show that abnormalities in the frontal lobes impact brain development and autism [20,21,22,23,24]. Until very recently, changing the folate status in humans has been shown to influence DNA methylation, but it was not established whether alteration in DNA methylation after changes in folate status are harmful in humans. However, in a challenging study, Wong *et al*., performed a genome-wide analysis of DNA methylation in a sample of 50 monozygotic (MZ) twin pairs sampled from a representatitive population cohort that included twins discordant and concordant for ASD, ASD-associated traits and no autistic phenotype [20]. Within-twin and between group analyses identified numerous differently methylated regions assocated with ASD. These researchers also indicated significant correlations between DNA methylation and quantatively measured autistic trait scores for the sample cohort. This is the first systematic epigenomic analysis of MZ twin discordant for ASD and implicates a role for the altered DNA methylation in autism. Findings by Wong and colleagues provide support for a potential role of DNA methylation via a high maternal folic acid intake in ASD and ASD-related traits [20].

## 4. Is Folic Acid Fortification Linked with Increased Prevalence of ASD?

Beginning 1 January 1998, FDA mandated fortification of manufactured cereal products went into effect and since then there has been a significant decrease in cases of neural defects in the U.S. [25,26]. Several researchers have questioned whether an increase in maternal folate early during pregnancy might be partly related to the unexplained increase in ASD cases in the U.S. It is interesting that closure of the neural tube and therefore its enablement by folic acid supplementation, occurs at a time during embryogenesis that is also critical in autism development [11,27,28,29,30]. It is well established that there has been a significant enhancement of maternal folate status since FDA mandated folic acid fortification of certain foods (1998) which has resulted in decreased incidence of NTDs during the mid-2000s [15]. This same time period coincides with the apparent beginning and continuous rise in the prevalence of autism and related disorders in the U.S. Investigators have wondered whether these similar time frames of change in maternal folate status and possible autism prevalence are a random event or that the improved maternal and resulting fetal folate status has played a role [11]. The enzyme dihydrofolate reductase (DHFR) is necessary to convert dietary folic acid to tetrahydrofolate before its one-carbon derivatives can be used in the body as coenzymes for various methylation reactions and nucleotide synthesis [15]. Bailey and Ayling have reported that the process of reduction of folic acid to tetrahydrofolate in humans is slow and highly variable [31]. They showed that in human liver, reduction of folic acid by DHFR on average was less than 2% of that of rat liver. Also, folic acid is an inhibitor of DHFR in reduction of its substrate 7,8 dihydrofolic acid. This limited ability to activate the synthetic vitamer raises questions about use of high levels of folic acid. Concern has been expressed that this unmetabolized folic acid may be detrimental [11]. Thus, it can be speculated that some mothers of children with ASD may have unusually low activity of DHFR.

Rogers and other researchers [11,28,29,30] have explored the possibility that a particular polymorphic form of the key enzyme methylenetetrahydratefolate reductase (MTHFR), required for activation of folate for methylation in neurodevelopment, exhibits reduced activity under low or normal folate levels but normal activity under higher folate nutritional status. In several studies, higher plasma homocysteine levels than in non-carriers, resulting from the presence of polymorphic forms of MTHFR during reduced or normal folate status have been shown to result in increased rates of miscarriages via thrombotic effects [28,30]. However, under the condition of enhanced folate status during the perinatal period, the incidence of hyper-homocysteinemia is reduced and thereby masks the latent adverse effects of the presence of this polymorphic form of MTHFR during pregnancy [32]. This polymorphism, although common in the normal population, is found with significantly higher frequency in children with autism [30]. Thus, it is hypothesized that enhanced folate status during pregnancy from fortification could have increased the survival rate of fetuses with genetic polymorphism such as MTHFR 667 C > T, which are associated with high homocysteine and subsequently require higher amounts of folate for the normal methylation needed for proper neurodevelopment [32]. Such polymorphisms have been observed in higher frequencies in children with autism, suggesting that these children might be genetically predisposed to less efficient folate metabolism and function [30,32]*.* Haggerty *et al*. [29] conducted a study to evaluate the concern that increasing folic acid intake through fortification may select for embryos with genotype that increase the risk of disease like autism in the offspring. They found no evidence to support that folic acid fortification or supplement use in pregnancy results in selection of deleterious genotype.

## 5. Decreased Risk of ASD with Improved Maternal Folate Status

Since folate and folic acid are essential for basic cellular processes, including DNA replication and protein methylation, it is biologically plausible that folic acid intake might affect numerous conditions positively or negatively depending on timing and dose. Several investigators have put forward hypotheses to explain the mechanism of association between folic acid and autism [33]. Ramaekers *et al*. [34,35] identified reduced 5-methylenetetrahydrofolate (5-Methyl THF) transport into the cerebrospinal fluid (CSF) in two autism spectrum disorders, *i.e.*, Rett syndrome and infantile low-functioning autism due to folate receptor autoimmunity. In spite of normal serum folate, CSF 5-Methyl THF was low in 23 of 25 patients and was explained by serum folate receptor autoantibodies (FRA) blocking the folate binding site of the membrane attached FR on the choroid epithelial cell. A partial or complete clinical recovery was reported after 12 months of oral folinic acid supplements in affected children. These researchers suggested that FR autoimmunity and cerebral folate deficiency appear to play a crucial role in the pathogenesis of autism spectrum disorders or in a particular subgroup of the autism spectrum [35]. In an open label study conducted by Frye *et al*., 93 children with autism also had a high prevalence (75.3%) of folate receptor antibodies (FRA) [36], In 16 children, the concentration of FRAs significantly correlated with cerebrospinal fluid 5-MTHFA, which were below the normative mean in every case. Children with FRAs were treated with oral leucovorin calcium (50 mg/day), and treatment response compared with the wait list control group. Compared to controls, significantly higher improvement ratings were observed in treated children over a 4 month period in verbal communications, receptive and expressive language and stereotypical behavior. This study further supports the role of folic acid as a risk factor for autism. Many recent studies have pointed to improved neurodevelopment in autistic children with mothers having higher folate concentrations or receiving folic acid supplements [10]. These studies conducted in America, Europe, Asia, and South Asia have shown consistent positive effects of maternal folate status in reducing the risk of autism in the offspring [9,37,38,39,40,41,42]. In an extensive study Adams *et al*. [37] compared the nutritional (vitamins, minerals and amino acids) and metabolic status (biomarkers of oxidative stress, methylation and sulfuation) of children with autism with that of healthy neurotypical children to evaluate the association of autism severity with nutrient biomarkers. Though plasma folic acid concentrations were not significantly different in autistic cases as compared to those of controls, a biomarker of functional need for folic acid, average FIGLU concentration, was significantly higher in children with autism as compared to neurotypical controls (1.99 µg/L ± 0.92 *vs.* 1.62 µg/L ± 072). Also, S-adenosyl methionine (SAM), the primary methyl donor in the body, was also significantly lower in children with autism (*p* < 0.001). James *et al*., conducted studies indicating higher vulnerability to oxidative stress and a decreased capacity for methylation which may contribute to development of autism [5,41]. In two studies, James *et al*., also reported significant improvement in transmethylation metabolites and glutathione redox status in autistic children after treatment for 3 months with oral supplements of 800 µg folinic acid and 1000 mg betaine twice/day and 400 µg of folinic acid twice/day and 75 µg/kg methyl B_12_ twice a week respectively [5,41].

Following the positive results associated with maternal folate status and reduced risk of ASD in various case-control studies, Suren *et al*., conducted an excellent epidemiologically sound study which confirmed the association between maternal use of prenatal folic acid supplements and subsequent decreased risk of ASD in children [8]. Suren *et al*., evaluated 85,176 children from the Norwegian Mother and Child cohort Study (MoBa) and reported an incidence of ASD of 0.10% in offspring of mothers who took periceptional folic acid supplements as compared to 0.21% in offspring of those who did not. No foods were fortified with folic acid at the time of recruitment of subjects for this study; therefore synthetic supplements represented the only source of folate other than that from the diet for the pregnant women. The subjects for this study kept a record of intake of vitamins, minerals, and other supplements as listed on the ingredient lists on the supplement containers within 4 week intervals from the start of pregnancy. In addition, to quantify supplement use and dietary intake, a food frequency questionnaire was administered at mid-pregnancy. This study has several strengths such as prospective design, use of validated instruments to collect data on supplement use during pregnancy, and active screening of children for autism and other neurodevelopmental disorders [8]. A limited evidence of selection bias was indicated by comparing their results in MoBa with Norwegians Medical registry data on risk of autistic disorder in folic acid supplement users *vs.* non-users and finding similar results. Furthermore, the prevalence of ASD was lower in MoBa than in the U.S. but similar to the prevalence in Norway. Beaudet [43] in response to discovery of inborn errors of metabolism associated with autism discussed the possibility of preventable forms of autism. Folic acid supplementation might correct or ameliorate underlying genetic variation in children, their parents, or both might drive the observed reduction in risk reported by Surin *et al*. [8]. Such genetic variation could include alterations in epigenetic regulator genes and their targets, which have been previously associated with risk of ASD.

Several epidemiological studies have tested the above mentioned hypotheses regarding the association between folic acid/folate and ASD. In this section studies are reported in chronological order and by study design. Relatively recent studies (starting in 2000) with well-designed methodology are included to examine the evidence for the involvement of folic acid/folate or alteration in folate metabolism and risk of ASD. A summary of these studies is presented in Table 1 [44]. With the exception of a prospective study in a Norwegian children cohort [8] and an open label trial [41] most of these investigations consist of case control designs, where maternal perinatal folate or multivitamin supplementation of children with ASD was retrospectively compared with maternal perinatal folate or multivitamins supplement use by healthy children.. A few investigators have also evaluated the effects of folate metabolites and their possible role as oxidative stressors as a risk factor for autism and the effect of interaction between maternal folate status and maternal genotype and risk of autism [12,28,39]. Results of these studies indicate an association between maternal perinatal folate status and ASD. Significant interaction effects have been reported for maternal MTHFR 677 TT, CBSrs234715 GT +TT, and child COMT 472 AA genotype, with greater risk for autism when mothers did not report taking prenatal vitamins peri-conceptionally. Schmidt *et al*. [9] have observed greater risk for children whose mothers had other one-carbon metabolism pathway gene variants and no maternal vitamin intake. As stated earlier, most of these investigations were retrospective case control studies with possibilities of various types of biases including differential misclassification of disease or/and exposure.

Currently several randomized clinical trials are underway to clarify and confirm the association between periconceptional folic acid intake and autism [45,46]. The Chinese Children and Family Study will evaluate potential benefits and adverse effects of periconceptional folic acid supplements in a 15 year follow-up of offsprings and mother [46]. In another open label clinical trial, with 40 autistic children, efficacy of methylcobalamine and folinic acid supplements would be determined. Whether treatment with these metabolic precursors would improve plasma bio-makers of oxidative stress and measures of core behaviors will be evaluated [45].

**Table 1:** Association between maternal folate intake, folate/folate metabolites, gene polymorphism and autism spectrum disorders [44].

Authors, Country and Year of Publication	Study Design and Subjects	Case Definition	Outcome Measure	Results
**M. Boris *et al.* U. S., 2004 [33]**	**Case Control Study** *Cases:* 168 Caucasian children 148 (84.5%) males 26 (5.5%) females *Controls:* 5389 Caucasian children	73.8% autism, 26.3% PDD diagnosed by neurologist, psychiatrist or neuropsychologist. DSM IV criteria used for diagnosis	Frequency of MTHFR alleles 677C*→ T* 1298A*→ C* in cases and controls	Significantly increased (*p* < 0.0001) Frequencies of homozygous mutation 677CT allele (23% in cases *vs.* 11% in controls) and heterozygous 677CT allele in cases (56%) *vs*. 41% in controls). Overall increased risk of ASD associated with common mutations affecting the folate/methylation cycle.
**S. J. James *et al*., U.S. 2006 [5]**	**Case Control study** *Cases:* 80 Caucasian children from autism clinics 89% males 11% females Mean age 7.3 ± 3.2 yreas *Controls:* 73 unrelated healthy Caucasian from a metabolic study Mean age:10.8 ± 4.1 years	Autism diagnoses: by independent specialists DSM IV or ADOS or CARS criteria	Plasma levels of folate metabolites: Methionine, Homocysteine Cystathionine, Cysteine SAM, SAH Glutathione Allele frequency: RFC 80 > A TCN2 776G > C COMT 472G > A MTHFR 667 > T	Plasma methionine and SAM/SAH ratio was significantly lower in autistic cases as compared to age matched controls (an indicator of lower methylation capacity). Antioxidant capacity as indicated by plasma levels of cysteine and glutathione was significantly decreased in cases. Significant increase in odds ratio, allele frequency and genotype distribution among autistic cases were found for: RFC-1 80A > G , TCN2 776C > G and COMT 472G > A genes An increase in frequency of MTHFR 667C > T reached border line significance in autistic cases. Increased vulnerability to oxidative stress related to folate metabolism may contribute to development of autism.
**V. T. Ramaekers *et al.* Belgium 2007 [34]**	**Case Control study**. *Cases:* 25 children with early-onset low functioning autism **Treatment:** !2 months of oral folinic acid supplementation (1.0 mg/kg/day) *Controls:* 100 healthy age matched controls	Diagnosis established by DSM IV criteria and ADOS in conjunction with ADI around 3 years of age	Serum folate, cerebrospinal (CSF) folate, CSF 5MTHF folate receptor (FR) autoantibodies	There was no significant difference in serum folate levels between autistic cases and controls. The mean CSF level of MTHF was significantly lower than controls (27.3 nmol/L compared to 82.0 nmol/L). Autistic cases with low CSF MTFR had autoantibodies of blocking type against human FR. The reduced CSF folate in autistic cases was associated with FR autoantibodies blocking the folate binding site. In cases, !2 months of oral folinic acid supplementation (1.0 mg/kg/day) resulted in normal CSF MTFR levels (75.5 nmol/L) and partial or complete clinical recovery.
**S.J. James *et al.* U.S. 2009 [41]**	**Open Label Trial** *Cases:* 40 autistic children 33 (82.0%) males 7 (18.0%) females Mean age: 4.8 ± 0.8 years. **Treatment***::* Treated with 400 µg follinic acid (twice/day) and 75 µg/kg methyl B_12_ (twice/week) for 3 months. *Controls:* 42 healthy age matched children Mean age: 4.5 ± 0.9 years	Autism diagnoses: by DSM IV and CARS score > 30	Metabolites in the transmethylation/transsulfuration pathway were measured before and after treatment and compared to values measured in controls	Pretreatment metabolite levels in autistic children were significantly different from values for controls. Intervention resulted in: significant increases in cysteine, cysteinylglycine and glutathione concentrations (*p* < 0.001) Glutathione redox ratio increased (*p* < 0.008) Mean metabolite concentration significantly improved after treatment but were below those of controls
**J.B. Adams *et al*., U.S. 2011 [37]**	Case control study Cases: 55 children with ASD 49 (89.0%) males 6 (11.0%) females Mean age:10 ± 3.1 years *Controls:* 44 neurotypical, non-sibling children of similar gender and geographical distribution Mean age:11 ± 3.1 years	ASD diagnoses: by Pervasive Developmental Disorder Behavior Inventory (PPDBI), Autism Treatment Evaluation Checklist (ATEC), Severity of Autism Scale (SAS)	Measurement of vitamins, biomarkers of vitamin status, minerals ,amino acids, plasma glutathione, and biomarkers of oxidative stress , methylation, sulfation and energy production	Mean serum levels of folic acid were lower in children with ASD than controls but were not significantly different. Biomarker of functional need for folic acid, urinary FIGLU was significantly higher in cases as compared to controls (1.99 µg /L *vs.* 1.66 µg /L) *p* = 0.03. Metabolite markers, plasma glutathione redox ratio and red blood cell SAM/SAH ratio were significantly higher in cases than controls (*p* < 0.0001 and *p* = 0.006)
**R.J. Schmidt *et al*., U.S., 2011 [39]**	**CHARGE population based Case Control Study** *Cases:* 284 autistic and 141 children with ASD between ages 2–5 years *Controls:* 278 children with typical development	Diagnoses confirmed by: Autism Diagnostic Interview-Revised and Autism Diagnostic Observation Schedule Generic	Consumption of prenatal multivitamins, nutrient specific vitamins at any time during the period 3 months before conception through pregnancy Maternal , paternal and child samples were genotyped for: MTHFR 667C > T MTHFR A1298C COMT 472G > A MTRR A66G TCN2	Use of prenatal vitamins during 3 months before and first month of pregnancy was associated with reduced risk of autism. (OR = 0.62, 95%.C.I.= 0.42–0.93) No association observed for vitamins intake during months 2 through 9 of pregnancy. Significant interactions were observed for autism between lack of perinatal maternal prenatal vitamin intake and both maternal MTFHR 667TT and CBSrs 234715GT+TT gentotypes. With combined OR = 4.5 , C.I. = 1.4–14.6 and 2.6 (1.2–5.4) respectively
**R.J. Schmidt *et al*., U.S., 2012 [9]**	**CHARGE Case Control study** *Cases:* 429 children with ASD *Controls:* 278 children with typical development. Cases and controls were frequency matched to age and catchment area, distribution of autism cases with a 4:1 male-to-female ratio	The diagnoses of autism cases were confirmed by the Autism Diagnostic Interview-Revised (ADI-R) and by ADOS. The two initially identified subgroups, ASD and Autism were later collapsed and results presented for combined ASD groups	Maternal folic acid intake was calculated from data on intake of multivitamins and prenatal vitamin including folic acid specific vitamins, cereal and supplements during the index period (3 months before and throughout pregnancy) Maternal and cases MTHFR 667C > T was genotyped	The mean (±SEM) folic acid intake was significantly greater for typical development children than for mothers of children with ASD (*p* < 0.01) in first month of pregnancy. A mean folic acid intake of ≥ 600 µg *vs.* <600 µg was associated with reduced risk of ASD (AOR = 0.62, 95% C.I. = 0.42–0.92) The association between folic acid and reduced ASD risk was strongest for mothers and children with MTHR 667C > T variant genotype. Periconceptional folic acid may reduce ASD risk in those with inefficient folate metabolism.
**V. T. Ramaekers *et al*., Belgium 2013 [35]**	**Case control Study** Cases: 75 children with infantile autism Controls: Non-autistic children with developmental delay	Diagnosis established by DSM IV criteria	Serum FR blocking Autoimmune antibodies	Blocking FR antibodies in cases had significantly higher prevalence (57%) than in non-autistic developmentally delayed children (33%) Mean serum folic acid levels 11.3 ng/mL) were significantly higher in cases than controls (9.30 ng/mL)
**P. Suren *et al*., Norway, 2013 [8]**	**Population based prospective study.** The study sample of 85,175 children born in 2002-2008 was derived from Primary Norwegian Mother and Child Cohort study (MoBa), with the mean age of 6.4 years Exposure of interest was use of folic acid from 4 weeks before to 8 weeks after the start of pregnancy.	Cases of ASD confirmed by linkages to the Norwegian Patient Registry, capturing data for all children diagnosed with ASD by Norwegian Health Services	Specialist confirmed diagnoses of ASD	270 children in the study sample were diagnosed with ASD at the end of the study. In children whose mother took folic acid, 0.10% had ASD as compared to 0.21% in those unexposed to folic acid. The AOR for autistic children of folic acid users was 0.61, 95% C.I. = 0.41–0.90 as compared to folic acid non-users. There was no association with Asperger syndrome or PDD-NOs, but power was limited. Use of prenatal folic acid supplements around the time of conception was associated with a significantly lower risk of ASD in the MoBa cohort.

Note: Enzymes and their alleles-MTHFR: 5,10-methylanetetrahydrofolate reductase, COMT: catechol-O-methyltransferase, MTR: methyltetrahydrofolate homocysteine methyl transferase, TCN2: transcobalamine II, CBS: cystathionine β-synthase.

## 6. Conclusions

Whether perinatal folic acid supplementation can prevent autism is still an open question. Results of several recent studies, including the prospective study by Surin *et al*. [8] are encouraging, but it is too early to say that universal periconceptional use of folic can reduce the incidence of ASD resulting from abnormal folate- methionine metabolism. Some very recent epigenetic studies in monozygotic twins provide support for potential role of DNA methylation via a high maternal folic acid intake in ASD [19,20]. Specifically, findings by Wong and colleagues [20] are provocative and provide support for a potential role of DNA methylation via a high maternal folic acid intake in ASD and ASD-related traits. It will be useful to measure the proportion of variance in autism explained by maternal folic acid status after adujusting for other known risk factors, particularly, in women who take periconception folic acid. Lack of definitive biological markers of autism for a majority of cases makes it difficult to evaluate the proportion of risk attributable to maternal folic acid intake. Moreover, the level of maternal folic acid intake which may result in sufficient cause to contribute to development of various forms of autism is difficult to isolate. Several randomized clinical trials with folinic acid supplementation are in progress and may provide some answers about whether folic acid supplementation has a protective or adverse role, if any, in relation to development of ASD.
